# Selective oversampling approach for strongly imbalanced data

**DOI:** 10.7717/peerj-cs.604

**Published:** 2021-06-18

**Authors:** Peter Gnip, Liberios Vokorokos, Peter Drotár

**Affiliations:** Department of Computers and Informatics, Technical University of Košice, Slovak Republic

**Keywords:** Imbalanced data, Oversampling, Outlier detection, SMOTE, ADASYN, Bankruptcy prediction

## Abstract

Challenges posed by imbalanced data are encountered in many real-world applications. One of the possible approaches to improve the classifier performance on imbalanced data is oversampling. In this paper, we propose the new selective oversampling approach (SOA) that first isolates the most representative samples from minority classes by using an outlier detection technique and then utilizes these samples for synthetic oversampling. We show that the proposed approach improves the performance of two state-of-the-art oversampling methods, namely, the synthetic minority oversampling technique and adaptive synthetic sampling. The prediction performance is evaluated on four synthetic datasets and four real-world datasets, and the proposed SOA methods always achieved the same or better performance than other considered existing oversampling methods.

## Introduction

Imbalanced data are ubiquitous in many machine learning application domains such as disease diagnostics (Wang et al., 2020), fraud and bankruptcy prediction (Somasundaram & Reddy, 2019; Zoričák et al., 2020; Le et al., 2019), software development (Yang et al., 2016) and many others (Haixiang et al., 2017). Imbalanced datasets represent data including samples that are not evenly distributed into different classes. The most frequently occurring scenario includes one class containing a majority of data samples while the samples in the other minority class are very rare. Conventional machine learning methods encounter difficulties in detecting these rare events because of their scarce occurrence (Thabtah et al., 2020). However, very often these rare events are of crucial importance. Rare events may be fraudulent financial transactions that would cause significant financial loss, or even the worst case: patients with some specific disease that would be diagnosed incorrectly and not receive proper treatment. It is very common in the medical domain that data from healthy patients represents the majority of data samples and that the particular disease occurs only occasionally.

As we already indicated, the traditional classification models face several challenges when classifying imbalanced data. Traditional methods such as support vector machines, random forest or neural nets yield sub-optimal performance. The prediction accuracy for the majority class is usually very high: however, the minority samples are misclassified. This scenario creates another problem. The standard evaluation metrics tend to focus on evaluation of the models with respect to the most frequent cases. Therefore, a model that correctly classifies majority samples, but fails to predict the correct class for the minority class, would demonstrate high classification accuracy. If the evaluation criterion such as classification accuracy is used to lead the learning process of the classifier, it degrades the objective performance of the model (Haixiang et al., 2017; Thabtah et al., 2020). Moreover, when using conventional methods the minority class samples are treated as noise during the learning process.

Many methodological approaches have been developed in recent years to cope with the issue of imbalanced learning. In general, these can be divided into several categories: sampling methods, cost-sensitive methods for imbalanced learning, ensemble methods and various hybrid methods (He & Garcia, 2009).

The sampling approach aims to modify the dataset prior to learning. There are two main approaches: oversampling and undersampling. Oversampling appends data to original data and extends the size of the minority class. On the other hand, undersampling removes data from the original dataset and reduces the number of samples of the majority class. The undersampling poses an obvious disadvantage of throwing away a portion of the data that can potentially contain some useful information. Oversampling can lead to overfitting, so proper methodology for model evaluation and validation is of crucial importance (Santos et al., 2018). The most frequently employed solution is synthetic oversampling, particularly the synthetic minority oversampling technique (SMOTE) that was successfully applied on different datasets (Chawla et al., 2002). SMOTE generates new samples of the positive class by interpolating several existing data points from minority class. During the generation of synthetic samples, SMOTE takes into account neighboring data points that in some cases increase overlap between the classes(Wang & Japkowicz, 2004). Several methods based on the SMOTE algorithm have been proposed to overcome this limitation, such as LR-SMOTE (Liang et al., 2020) and ADASYN (He et al., 2008). ADASYN considers the complexity of samples when generating synthetic data. The more synthetic samples are generated for complex data samples that are harder to learn. On the other hand, LR-SMOTE focuses on samples close to the sample center to make sure that no outlier samples are generated and the dataset distribution is preserved.

The second popular approach is cost-sensitive learning. In cost-sensitive learning, the cost for misclassified minority samples is significantly higher than the cost for incorrectly classified majority samples. In contrast to sampling approaches, in cost-sensitive learning it is necessary to enhance the learning algorithm to take into account the cost matrix (Tao et al., 2019; Xiao et al., 2020). The cost-sensitive implementations of popular classifiers such as AdaBoost, decision trees and neural networks are discussed in (He & Garcia, 2009). Even though the cost-sensitive learners offer good performance in imbalanced scenarios, their utilization is quite limited due to their complexity of implementation for non-expert users and non-straightforward determination of cost matrix values.

The ensemble learning approach has been used for several years already(Kuncheva & Whitaker, 2003). Ensemble classifiers have proved their robust character and have been successfully applied to solve many challenging real-world machine learning tasks (Galar et al., 2011; Liu, Luo & Li, 2018). An ensemble classifier is built from multiple base classifiers by combining their individual decisions. For the class imbalance problem, these ensembles are built either as cost-sensitive boosting ensembles or boosting-based ensembles with embedded data pre-processing (Galar et al., 2011).

In this paper, we focus on the data oversampling approach since it is independent of the selected classifier and can be used as a form of pre-processing for any imbalanced dataset. We build upon existing SMOTE and ADASYN oversampling techniques and propose the novel data oversampling approach to further enhance the performance of these techniques. In our approach, we employ an outlier detection technique, namely one-class support vector machine, to selectively choose samples that are used for generation of synthetic samples. Samples not selected by outlier detection, i.e., samples resembling the majority class, are not oversampled. We show that the proposed approach results in improved prediction based on artificial and real-world data.

The rest of the paper is organized as follows. In the next section we describe the proposed selective oversampling approach. Then, we study the behavior of SOA by analyzing the data after oversampling and evaluate the performance on synthetic and real-world datasets. Finally, we draw conclusions and outline the future work.

## Proposed Approach

In this section, we describe the proposed selective oversampling approach (SOA). SOA combines an outlier detection technique with oversampling in order to obtain a more descriptive training dataset before applying the learning algorithm.

### Selective Oversampling Approach

To explain the essential idea of SOA, let us first define an imbalanced dataset *D*_*imb*_** that consists of *n* observations defined as {*x*_*i*_, *y*_*i*_}**, where *i* = 1, 2, …, *n* and *y*_*i*_ = {0, 1}** expresses a corresponding class of binary classification task. We assume that the original dataset is divided into training subset (*D*_*train*_**) and testing (*D*_*test*_**) subset.

First, SOA utilizes an outlier detection technique that is trained only on data samples from the majority class. We selected one-class support vector machines (OCSVM) as an outlier detection method since it provides satisfactory results in a wide area of applications. The trained outlier detection model is applied to minority class samples and all misclassified observations are removed from the training dataset. Our assumption is that by selecting only correctly identified minority samples, we are selecting the most representative samples of the minority class. Minority samples that were misclassified by outlier detector probably do not share the characteristics of the minority class. As such, when oversampled, they can mislead the classifier. Further processing is performed only on minority class samples that were identified as outliers. The outlier detection model treats minority samples as outliers.

**Figure 1:**
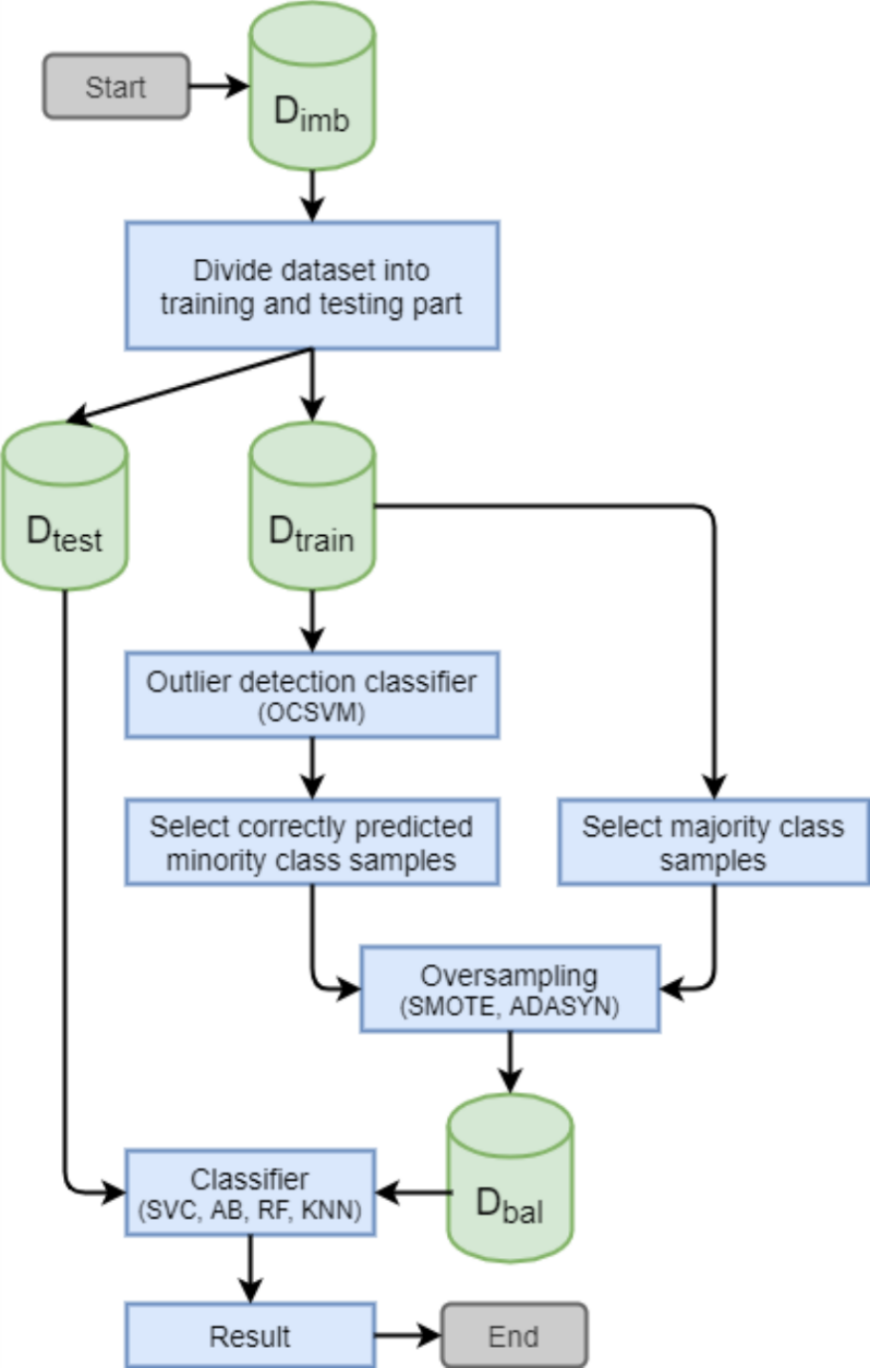
Principle of the proposed selective oversampling approach.

The second step is to balance the distribution of the training dataset (*D*_*bal*_**) by generating synthetic minority class samples. Different oversampling methods can be used for this purpose. In this study, we compare the utilization of two oversampling methods: SMOTE and ADASYN. The selective oversampling approach based on SMOTE is denoted as SOA-S and the approach based on ADASYN is denoted as SOA-A. The general principle of the proposed SOA is depicted in Fig. 1.

### One-class support vector machine

We employ OCSVM to select the most representative data points from minority samples. OCSVM is an unsupervised outlier detection classifier based on Vapnik’s well-known idea about support vector machines (Vapnik, 2013). The initial assumption is that outliers (minority class samples) occupy the low density regions of the data feature space and that the kernel model is able to recognize high density regions (majority class samples). The goal objective is to find an optimal decision function *f* that is able to identify outliers by mapping the data samples into feature space *F* and separating them from the origin with maximum possible margin (Schölkopf et al., 2001). This can be achieved by solving the quadratic programming task.

Assume a training dataset *x*_1_, *x*_2_, …, *x*_*n*_ ∈ *X* where *n* ∈ *N* represents the number of samples. Additionally, let Φ** be a feature map *X* → *F* that maps observations from *X* into inner product feature space *F*. The image of the map function Φ** is computed by evaluating the kernel and is formulated as

(1) \begin{eqnarray*}k(x,y)=(\Phi (x)\ast \Phi (y)).\end{eqnarray*}

The goal objective is to separate data samples from the origin via hyperplanes. This can be achieved by solving the quadratic programming task defined as

(2) \begin{eqnarray*}\min _{w\in F,\xi \in {R}^{n},\rho \in R} \frac{1}{2} {|}{|}w{|}{{|}}^{2}+ \frac{1}{{v}^{n}} \sum _{i}{\xi }_{i}-\rho \end{eqnarray*}

\begin{eqnarray*}subject~to(w\ast \Phi ({\mathbi{x}}_{i}))\geq \rho -{\xi }_{i},{\xi }_{i}\geq 0, \end{eqnarray*}

where parameter *v* expresses the fraction of support vectors and anomalies, *ξ*_*i*_** is a slack variable and *ρ* is an offset parameterizing a hyperplane in the feature space. Moreover, the output of the following decision function

(3) \begin{eqnarray*}f(x)=sgn((w\ast \Phi ({x}_{i}))-\rho ).\end{eqnarray*}

is positive for the most observations *x*_*i*_** if the result of the quadratic programming task is 1 and the regularization term ||*w*||** is reaching a small value. By introducing Lagrange multiplier method and using multipliers *α*_*i*_, *β*_*i*_ ≥ 0**, the above optimization problem can be solved by its dual form

(4) \begin{eqnarray*}min_{\alpha } \frac{1}{2} \sum _{ij}{\alpha }_{i}{\alpha }_{j}k({x}_{i},{x}_{j})\end{eqnarray*}

\begin{eqnarray*}where~0\leq {\alpha }_{i}\leq \frac{1}{{v}^{l}} ,\sum _{i}{\alpha }_{i}=1. \end{eqnarray*}

Then, the offset *ρ* parameterizing a hyperplane in the feature space associated with the kernel is defined as follows

(5) \begin{eqnarray*}\rho =(w\ast \Phi ({x}_{i}))=\sum _{j}{\alpha }_{i}{\alpha }_{j}k({x}_{i},{x}_{j}).\end{eqnarray*}

### Generation of synthetic samples

In general, any oversampling method can be used at this stage. We employ two state-of-the-art oversampling techniques: SMOTE and ADASYN. SMOTE is the most famous method for data oversampling. Even though it has some shortcomings it performs satisfactory in many applications (Fernández et al., 2018). ADASYN build upon the SMOTE but put more focus on minority class samples that are more difficult to learn. ADASYN showed improvement in performance over SMOTE in some domains (He et al., 2008).

#### SMOTE

SMOTE is a data oversampling technique used for generating samples from the minority class in order to acquire a class-balanced or nearly class-balanced training dataset. The essential idea behind the SMOTE algorithm is to derive new synthetic samples using existing minority class samples rather than by oversampling with replacement, which often does not improve the classifier ability to recognize the minority class observations (Chawla et al., 2002). In our approach, we use only a subset of minority class samples to generate synthetic data.

The SMOTE algorithm determines the number of artificial samples that are to be generated to obtain the desired class-balance level of the training dataset. Each artificial sample *x*_*a*_** is generated as a linear interpolation of two similar minority class samples. This can be expressed as

(6) \begin{eqnarray*}{x}_{a}={x}_{i}+random(0,1)\times ({x}_{i}-{x}_{k}),\end{eqnarray*}

where *x*_*k*_** is the randomly selected minority class sample from *k*-nearest neighbors to sample *x*_*i*_** calculated by Euclidean distance metric.

#### ADASYN

ADASYN is a data sampling technique used for balancing the skewed class distribution. The main idea of the ADASYN is to generate synthetic minority class samples with emphasis on samples that are harder to detect. The strategy proposed by He et al. (2008) is to use a density distribution *r*_*i*_** as a criterion to automatically decide the number of samples that is required to be generated for each observation from the minority class.

Let *n*_*minority*_** be the number of minority class samples and *n*_*majority*_** be the number of majority class samples. The ADASYN algorithm starts with evaluation of class imbalance degree, which is defined as

(7) \begin{eqnarray*}d= \frac{{n}_{minority}}{{n}_{majority}} .\end{eqnarray*}

If *d* is lower than the actual maximum tolerated threshold for class imbalance degree, the number of synthetic data samples that needs to be generated is calculated. It is expressed by

(8) \begin{eqnarray*}G=({n}_{minority}-{n}_{majority})\times \beta ,\end{eqnarray*}

where *β* ∈ [0, 1]** specifies the expected balance level after resampling. In a further step, the density distribution *r*_*i*_** is calculated for each observation *x*_*i*_** belonging to the minority class using the following equation

(9) \begin{eqnarray*}{r}_{i}= \frac{{\Delta }_{i}}{k} subject to i=1,2,\ldots ,{n}_{minority}.\end{eqnarray*}

The *k* and Δ_*i*_** parameters define the number of the nearest neighbors to sample *x*_*i*_** of the minority and majority class, respectively. The most similar samples are identified by Euclidean distance metric. The density distribution can also be expressed by its normalized form, defined as follows

(10) \begin{eqnarray*}{\hat {r}}_{i}= \frac{{r}_{i}}{\sum _{i=1}^{{n}_{minority}}{r}_{i}} .\end{eqnarray*}

The final step is to calculate the number of required synthetic data samples *g*_*i*_** that need to be created for observation *x*_*i*_**. This is defined by the following equation

(11) \begin{eqnarray*}{g}_{i}={\hat {r}}_{i}\times G.\end{eqnarray*}

After that, each synthetic sample *s*_*i*_** for observation *x*_*i*_** is generated using the following formula

(12) \begin{eqnarray*}{s}_{i}=({x}_{zi}-{x}_{i})\times random(0,1),\end{eqnarray*}

where (*x*_*zi*_ − *x*_*i*_**) specifies the difference vector for a randomly chosen sample from the *k*-nearest neighbors and sample *x*_*i*_**.

## Computer Experiments

In this section, we provide brief characteristics of the acquired datasets, their visualizations after oversampling and a description of the base classifiers utilized during the decision-making process. The performance of proposed SOA-S and SOA-A methods is compared to two state-of-the-art oversampling methods: SMOTE and ADASYN. We present methodology and discuss the evaluation of prediction performance.

### Data

During the computer experiments, eight different imbalanced datasets representing binary classification problems were utilized. We used four slightly different artificial datasets (*Synt. dataset-1*, *Synt. dataset-2*, *Synt. dataset-3* and *Synt. dataset-4*) and four real-world datasets for comparison (*Bankruptcy - manufacture*, *Bankruptcy - construction*, *Wine* and *Bank marketing*).

*Synt. dataset-1*, *Synt. dataset-2*, *Synt. dataset-3* and *Synt. dataset-4* were artificially generated via Scikit-learn library generators (Pedregosa et al., 2011) with the following number of observations 2,200, 1,500, 1,500 and 2,414, respectively. In those datasets, the features are situated on the vertices of a five-dimensional hypercube or drawn randomly from a Gaussian distribution. We have used a slightly modified Madelon dataset (Guyon, 2003) that represents imbalanced class distribution. The significant difference between artificially generated datasets is the class imbalance level, being 20:1 for *Synt. dataset-1*, 39:1 for *Synt. dataset-2*, 50:1 for *Synt. dataset-3* and 70:1 for *Synt. dataset-4*. We also utilized a different number of features, 20 features for *Synt. dataset-2* and *Synt. dataset-3* and 40 features for *Synt. dataset-1* and *Synt. dataset-4*.

The *Bankruptcy - manufacture* and *Bankruptcy - construction* datasets consist of thousands of annual reports of *SMEs* companies operating in the manufacture and construction business area in the Slovak Republic during the year 2015. The company data are represented by 20 financial attributes. Minority class samples represent financially distressed (bankrupt) companies while majority class samples stand for solvent (non-bankrupt) companies. These are part of the larger dataset described in Drotár et al. (2019). The *Bank marketing* dataset is related with direct marketing campaign of a Portuguese banking institution based on a phone calls. The aim was to asses whether the bank term deposit would be subscribed or not. This dataset is a subset of a larger dataset described in Moro, Cortez & Rita (2014). The *Wine* dataset consists of taste preferences of white wine samples from the north of Portugal (Cortez et al., 2009). The wine quality data are represented by 11 attributes based on physicochemical tests. Here, the minority samples represent the high-quality wine with a quality score from 8 to 10 and the majority class samples represents low-quality wine with a quality score from 0 to 7. The overview of all datasets is depicted in Table 1.

**Table 1:** Detailed characteristic of utilized datasets.

Dataset	Samples	Attributes	Imbalance ratio	Reference
Synt. dataset-1	2200	40	20:1	Guyon (2003)
Synt. dataset-2	1500	20	39:1	Guyon (2003)
Synt. dataset-3	1500	20	50:1	Guyon (2003)
Synt. dataset-4	2414	40	70:1	Guyon (2003)
Bankruptcy - manufacture	5854	20	417:1	Drotár et al. (2019)
Bankruptcy - construction	3128	20	222:1	Drotár et al. (2019)
Wine	4898	11	26:1	Cortez et al. (2009)
Bank marketing	4119	20	8:1	Moro, Cortez & Rita (2014)

### Visualization of data distribution after oversampling

**Figure 2:**
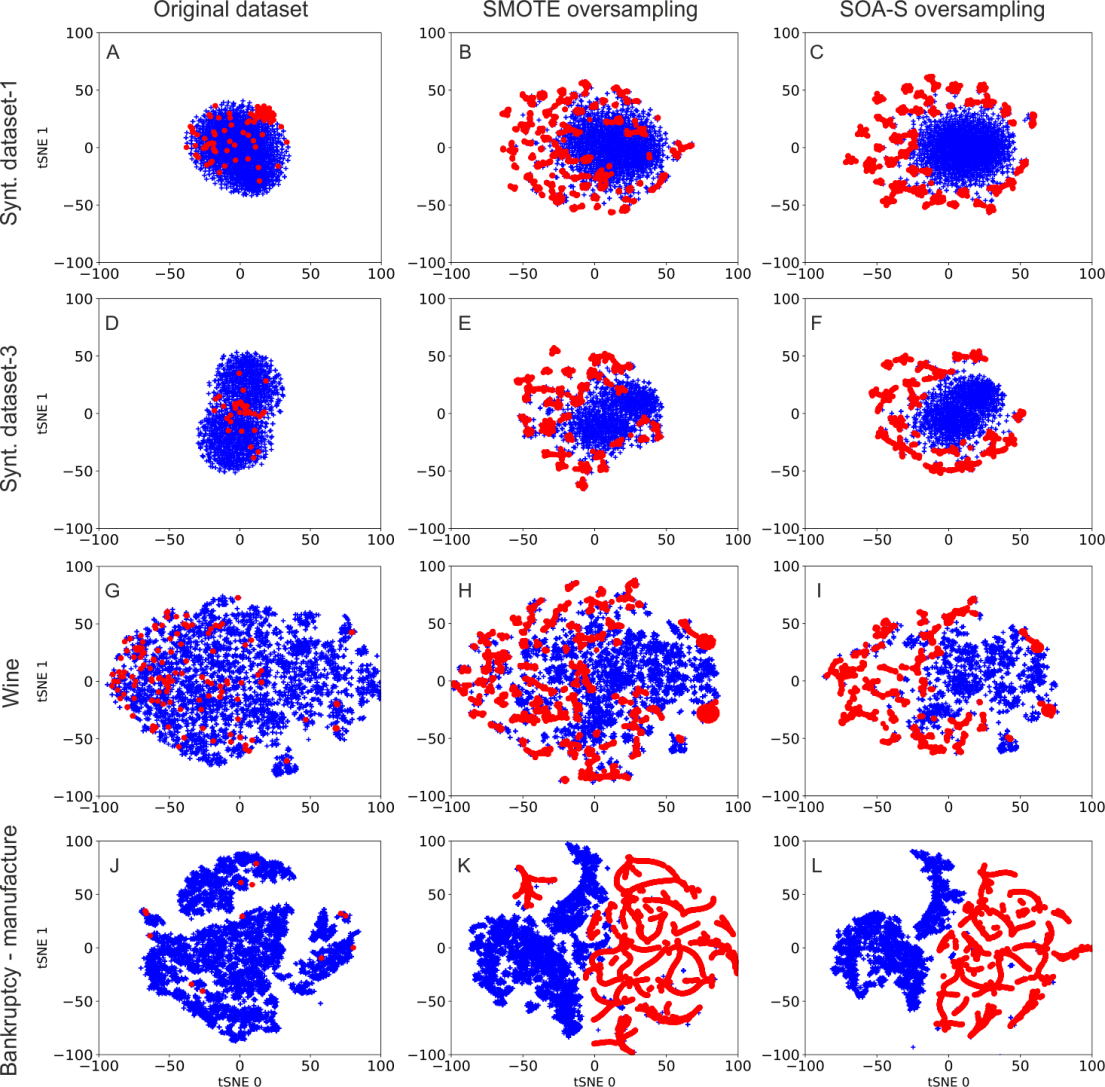
Visualization of data samples after applying SMOTE and SOA-S method in 2-dimensional space.

To obtain some overview about data distribution after oversampling, we visualize the distribution of original datasets and datasets after oversampling. We use the t-distributed stochastic neighbor embedding (t-SNE) method (Van der Maaten & Hinton, 2008). t-SNE is a state-of-the-art visualization method that converts high-dimensional data to two dimensions. It is based on nonlinear local relationship between the data points and performs transformation to two dimensional space in the way that more similar samples are modeled as nearby points and dissimilar samples are modeled as distant points in two dimensional space. t-SNE visualizations of selected four datasets are depicted in Fig. 2. The first column represents visualization of the original imbalanced data. The middle column shows the data after SMOTE oversampling. As seen, the SMOTE created clusters of data points that emerged after generation of synthetic samples. The data distribution after SOA-S oversampling is illustrated in the last column. The distribution of the data is similar to that of SMOTE oversampling. This is expected since the SOA-S is built on top of the SMOTE oversampler. However, there is one significant difference. The SOA-S oversampling results in better differentiability between two classes. As we can see, the minority class data (red color) are more separated from the other class data in the case of SOA-S oversampling.

### Evaluation of prediction performance

To evaluate the effect of oversampling, we use four different classifiers: support vector machines, k-nearest neighbors, AdaBoost and random forest. All classifiers are applied on four synthetic datasets and four real-world datasets as described in the previous sections. The brief description of utilized algorithms is as follows.

#### K-nearest neighbors

The k-nearest neighbors classifier or KNN is one of the most commonly used supervised and instance-based machine learning algorithms. The essential idea of the KNN algorithm is based on the assumption that the nearest observations to sample *x*_*i*_** for which we seek the label are the most representative ones, and the class label is assigned by simple majority voting of the nearest neighbors(Kramer, 2013). The final label of sample *x*_*i*_** is obtained by applying the following decision rule

(13) \begin{eqnarray*}f({x}_{i})= \left\{ \begin{array}{@{}ll@{}} \displaystyle 1 &\displaystyle if\sum _{c=1}^{{n}_{k({x}_{i})}}{y}_{i}\geq 0\\ \displaystyle -1 &\displaystyle if\sum _{c=1}^{{n}_{k({x}_{i})}}{y}_{i}\lt 0, \end{array} \right. \end{eqnarray*}

where *n*_*k*(*x*_*i*_)_** expresses the indexes of the k-nearest observations. The most similar samples are calculated by using various distance algorithms.

#### AdaBoost

AdaBoost (AB) is a supervised ensemble boosting algorithm that was proposed by Freund & Schapire (1997). The essential idea behind AdaBoost is based on combining multiple sequentially trained base classifiers in order to obtain a more powerful model with increased prediction performance versus each individually trained classifier. This is achieved by boosting weights of incorrectly predicted samples during the training process.

To explain the principle of the AdaBoost algorithm, let us assume a training dataset {*x*_*i*_, *y*_*i*_** } consisting of *n* observations. Additionally, let *n*_*base*_** be the number of base classifiers. The initial step starts with training of the base classifier using the equal weights distribution. During all subsequent training iterations, the higher weights are reassigned to each incorrectly predicted sample in order to increase the probability for correct classification. This training process is repeated until all training samples are correctly classified or the stopping criterion is reached. Afterwards, the final model *C*(*x*)** is formed as a linear combination of base classifiers *c*_*j*_**. It is defined by the following equation

(14) \begin{eqnarray*}C(x)=\sum _{j=1}^{{n}_{base}}{w}_{j}{c}_{j}(x),\end{eqnarray*}

where *w*_*j*_** expresses the distribution of weights for a particular base classifier.

#### Random forest

The random forest (RF) is a supervised ensemble machine learning algorithm introduced by Breiman (2001). The main idea is based on combining the outputs of multiple tree predictors(Decision Tree classifier) using so-called bootstrap aggregating technique Breiman (1996). This technique improves prediction performance of base learners and controls overfitting. In this case, the RF classifier is the final class label for observation *x*_*i*_** derived from the output of each individually trained base classifier using the majority voting function formulated as follows

(15) \begin{eqnarray*}f({x}_{i})= \frac{1}{n} \sum _{c=1}^{n}{f}_{c}({x}_{i}),\end{eqnarray*}

where *f*_*c*_(*x*_*i*_)** indicates the output label of each individually trained base classifier. The decision-making process depends on the ability to learn decision rules contained in the dataset. The label with the most votes is assigned to a particular sample.

#### Support vector machines

The support vector machine or SVC algorithm is a well-known and widely used supervised machine learning method based on the famous idea of support vector machines Vapnik (2013). The strategy behind the SVC algorithm is to construct an optimal decision boundary with maximum possible margin using a nonlinear mapping of data samples into high-dimensional feature space, which also enables classification of non-linearly separable observations by using the so-called kernel trick (Hearst et al., 1998). The optimization problem solved by SVM has been outlined in section II.B.

### Numerical results

All datasets were standardized per feature to have zero mean and unit variance. We used five-fold stratified cross validation to validate the model. The experiments were performed 50 times and the results were averaged. To tune the classifier performance, we searched through the grid of hyperparameters. For SVC, we searched the grid of hyperparameters defined by *γ* = [0.001, 0.01, 0.1, 1, 10, 100] and *C* = [0.01, 0.1, 1, 5, 10, 100]. In case of AB, we changed the number of estimators from 50 to 500 with step 50. Similarly, in RF we searched through number of estimators *N*_*est*_, with max depth of three *L*_*tree*_ and maximal number of features *N*_*feats*_, where *N*_*est*_ = [100, 200, 300, 400], *L*_*tree*_ = [100, 200] and N_feats_= [’auto’, ’log2’]. Finally, in the case of KNN we searched the space defined by number of neighbors *N*_*N*_ = [5, 7, 10], leaf size *L* = [30, 50, 100, 100] and considered both Minkowski and Euclidean distance.

Choosing the right evaluation metric while working with imbalanced data is one of the most crucial steps. The prediction performance of classification models was measured by geometric mean (GM). The GM is considered as one of the most reliable techniques while working with imbalanced data(Helal, Haydar & Mostafa, 2016). It is expressed as the square root of the product of sensitivity and specificity and is defined as follows

(16) \begin{eqnarray*}GM=\sqrt{SENSITIVITY\times SPECIFICITY}.\end{eqnarray*}

Sensitivity represents the proportion of actual positive cases that have been predicted as positive by our model, and it is expressed as

(17) \begin{eqnarray*}SENSITIVITY= \frac{TP}{TP+FN} .\end{eqnarray*}

TP (*True Positive*) is the number of correctly predicted positive cases and FN (*False Negative*) is the number of incorrectly predicted positive cases. The proportion of correctly predicted negative cases is expressed by specificity and is defined as follows

(18) \begin{eqnarray*}SPECIFICITY= \frac{TN}{TN+FP} ,\end{eqnarray*}

where TN (*True Negative*) expresses the number of correctly predicted negative cases and FP (*False Positive*) expresses the number of incorrectly predicted negative cases.

The prediction accuracy of all four classification models trained on synthetic datasets is depicted in Table 2. As expected, the application of classifiers without the use of any sampling technique resulted in poor model performance. As the imbalance ratio of the dataset increases, performance of the models trained on non-oversampled data sharply decreases. For the *Synt. dataset-4* the GM score of models without oversampling was less than 40%. This was probably caused because the model’s assumption about the equal data distribution resulted in bias towards the majority class while facing imbalanced data. Significant improvement was achieved by applying SMOTE and ADASYN sampling methods to acquire a class-balanced dataset that considerably increased prediction performance of each individual classifier. Again, the performance gains for datasets with smaller imbalance ratio are smaller than for those with high imbalance ratios. The best results were yielded by the SVC classifier, with GM score between 72% to 87%. Applying SOA-S and SOA-A as data balancing approaches further improved performance of all employed classifiers. Again, the best results were obtained by SVC, followed by the AB classifier. The SOA methods outperformed SMOTE and ADASYN in majority of cases. The only exception was result of SVC classifier on *Synt. dataset-1*. The SVC based on SMOTE oversampling data slightly outperformed SOA. This may indicate that the SOA is better suited for datasets with higher imbalance ratio. This is confirmed by increasing performance gains of SOA methods when the imbalance ratio of dataset is higher.

**Table 2:** The best *GM* scores (%) achieved on the synthetic datasets (± for standard deviation).

Dataset	Sampling	SVC	AB	KNN	RF
Synt. dataset-1	none	66.73 ± 13.5	75.41 ± 10.1	33.14 ± 15.3	70.42 ± 16.6
	SMOTE	**87.01 ± 4.07**	84.72 ± 5.62	78.61 ± 5.67	78.13 ± 11.3
	ADASYN	86.82 ± 4.13	84.93 ± 5.48	77.59 ± 5.53	78.04 ± 11.4
	SOA-S	86.94 ± 3.39	**86.79 ± 4.33**	**82.79 ± 4.64**	**80.02 ± 9.63**
	SOA-A	86.77 ± 3.61	86.77 ± 4.61	82.74 ± 4.49	79.79 ± 9.75
Synt. dataset-2	none	63.40 ± 17.5	69.81 ± 13.7	37.80 ± 22.1	66.06 ± 18.3
	SMOTE	85.73 ± 4.61	80.50 ± 6.83	79.69 ± 7.09	72.53 ± 12.9
	ADASYN	85.65 ± 4.82	80.57 ± 6.65	79.48 ± 6.86	70.89 ± 14.1
	SOA-S	**86.71 ± 3.82**	84.03 ± 5.22	81.99 ± 5.42	**73.20 ± 12.2**
	SOA-A	86.46 ± 3.99	**84.45 ± 5.33**	**82.08 ± 5.55**	73.17 ± 11.7
Synt. dataset-3	none	54.63 ± 20.5	61.75 ± 17.9	27.67 ± 22.6	54.07 ± 21.6
	SMOTE	82.57 ± 5.81	75.36 ± 9.85	76.13 ± 7.94	58.77 ± 18.4
	ADASYN	82.55 ± 6.15	75.34 ± 9.22	76.09 ± 8.49	57.56 ± 18.4
	SOA-S	**84.61 ± 4.32**	79.23 ± 6.19	**79.42 ± 6.36**	**60.71 ± 16.8**
	SOA-A	84.10 ± 4.65	**79.34 ± 6.89**	79.39 ± 6.27	60.28 ± 15.9
Synt. dataset-4	none	21.24 ± 24.6	36.32 ± 21.6	5.31 ± 14.9	20.88 ± 24.9
	SMOTE	72.94 ± 7.52	60.80 ± 9.85	67.72 ± 6.65	19.59 ± 20.9
	ADASYN	72.14 ± 7.78	61.32 ± 10.7	67.69 ± 6.29	17.95 ± 19.8
	SOA-S	**76.67 ± 5.29**	68.86 ± 8.09	74.84 ± 6.13	**29.38 ± 18.6**
	SOA-A	75.22 ± 7.78	**69.34 ± 7.33**	**74.99 ± 6.45**	28.09 ± 17.7

**Notes.**

Highest results are in bold.

To assess the effectiveness of proposed oversampling approaches on real-world data, we choose four imbalanced real-world datasets. The overview of achieved results is depicted in Table 3. As can be determined from the result of the classifiers without any oversampling technique, the selected datasets represent very challenging classification tasks. In case of two highly imbalanced bankruptcy datasets, the prediction performances of AB, RF and KNN, measured GM scores, are very low, even after oversampling the data with SMOTE/ADASYN. Only SVC can provide reasonable results. Similarly, as with case of synthetically generated datasets, the utilization of the proposed SOA methods also led to increased GM scores in the case of bankruptcy datasets. The *Wine* and *Bank marketing* datasets have class distribution 26:1 and 8:1, respectively. Application of SOA-S and SOA-A methods allowed for increase in prediction performance also in case of these two real-world datasets. Similarly as in the case of synthetic datasets, the best results were achieved by SVC classifier.

**Table 3:** The best *GM* scores (%) achieved on the real-world datasets ( ±  for standard deviation).

Dataset	Sampling	SVC	AB	KNN	RF
Bankruptcy - manufacture	none	7.44 ± 11.3	22.37 ± 12.7	5.65 ± 9.84	5.60 ± 9.93
	SMOTE	90.61 ± 2.36	52.70 ± 12.4	39.70 ± 11.3	12.33 ± 7.95
	ADASYN	90.65 ± 2.38	53.48 ± 12.3	62.79 ± 10.9	12.24 ± 7.92
	SOA+S	95.13 ± 1.09	**72.13 ± 11.8**	61.41 ± 10.7	55.37 ± 14.3
	SOA+A	**95.23 ± 1.14**	71.09 ± 12.1	**65.44 ± 9.52**	**53.53 ± 14.2**
Bankruptcy - construction	none	33.45 ± 13.2	44.46 ± 13.1	5.20 ± 9.74	14.54 ± 10.4
	SMOTE	94.54 ± 1.42	57.99 ± 14.8	62.19 ± 13.8	27.35 ± 12.9
	ADASYN	94.52 ± 1.33	61.35 ± 13.7	61.86 ± 13.3	28.91 ± 13.4
	SOA+S	**95.69 ± 0.96**	78.43 ± 10.7	**79.57 ± 8.51**	**59.83 ± 11.2**
	SOA+A	95.65 ± 0.99	**80.22 ± 10.1**	78.52 ± 9.93	55.56 ± 13.4
Wine	none	64.06 ± 2.45	48.41 ± 1.89	63.21 ± 2.37	60.82 ± 2.32
	SMOTE	77.49 ± 0.91	73.71 ± 1.21	79.28 ± 1.35	70.90 ± 2.33
	ADASYN	77.48 ± 1.01	73.86 ± 1.38	79.46 ± 1.28	71.27 ± 2.31
	SOA+S	**78.94 ± 0.74**	**77.91 ± 0.65**	81.21 ± 1.07	79.81 ± 0.87
	SOA+A	78.79 ± 0.69	77.76 ± 0.78	**81.24 ± 1.36**	**79.97 ± 0.79**
Bank marketing	none	64.69 ± 0.78	67.71 ± 0.82	54.06 ± 0.71	65.47 ± 1.04
	SMOTE	86.88 ± 0.28	81.99 ± 1.21	79.13 ± 0.67	73.84 ± 0.81
	ADASYN	87.02 ± 0.29	81.36 ± 0.93	79.23 ± 0.79	73.55 ± 0.83
	SOA+S	**87.04 ± 0.22**	**83.31 ± 0.52**	**79.74 ± 0.54**	83.38 ± 0.51
	SOA+A	87.02 ± 0.35	83.02 ± 0.48	79.42 ± 0.99	**84.19 ± 0.69**

**Notes.**

Highest results are in bold.

The proposed SOA employed in the first stage two data generation methods: SMOTE and ADASYN. It is interesting to note that the performance of the SOA is not significantly affected by the choice of the data generation strategy. Both SOA-S and SOA-A perform very similarly on all evaluated artificial and real-world datasets.

## Conclusion

The data imbalance represents an important issue in many machine learning applications. In this paper, we propose a selective oversampling approach that first isolates the most representative data points and than employs the oversampling strategy for minority class data. Our results showed that SOA-S and SOA-A methods outperformed SMOTE and ADASYN sampling techniques for all selected classifiers on four artificial and four real-world datasets. We have noticed that the oversampling performed much better in combination with some classifiers than with others. For the majority datasets the nonlinear SVM yielded the highest GM scores. In case of some datasets the difference between the classifiers was more than ten percent in performance. This is true for not-oversampled but also for oversampled data and for all oversampling techniques. Therefore we recommend to pay attention to the choice of the classifier for the oversampled data.

As a further work, we aim to investigate how oversampling can be adopted to provide more robust performance of the subsequent classifier and whether the choice of classifier can be determined a priori based on oversampled data distributions.

## Additional Information and Declarations

### Data Availability

The following information was supplied regarding data availability: The datasets (four artificially generated datasets and four real-world datasets) and source code are available at GitHub: https://github.com/peterG944/SOA.
